# Supplementary material for: Complex‐centric proteome profiling by SEC‐SWATH‐MS
Source: Mol Syst Biol. 2019 Jan 14;15(1):e8438. doi: 10.15252/msb.20188438 (PMC6346213; doi:10.15252/msb.20188438)
Supplement: Supplementary file 6 — Dataset EV5 [file MSB-15-e8438-s006.zip › feature_plots_corum/643.pdf]

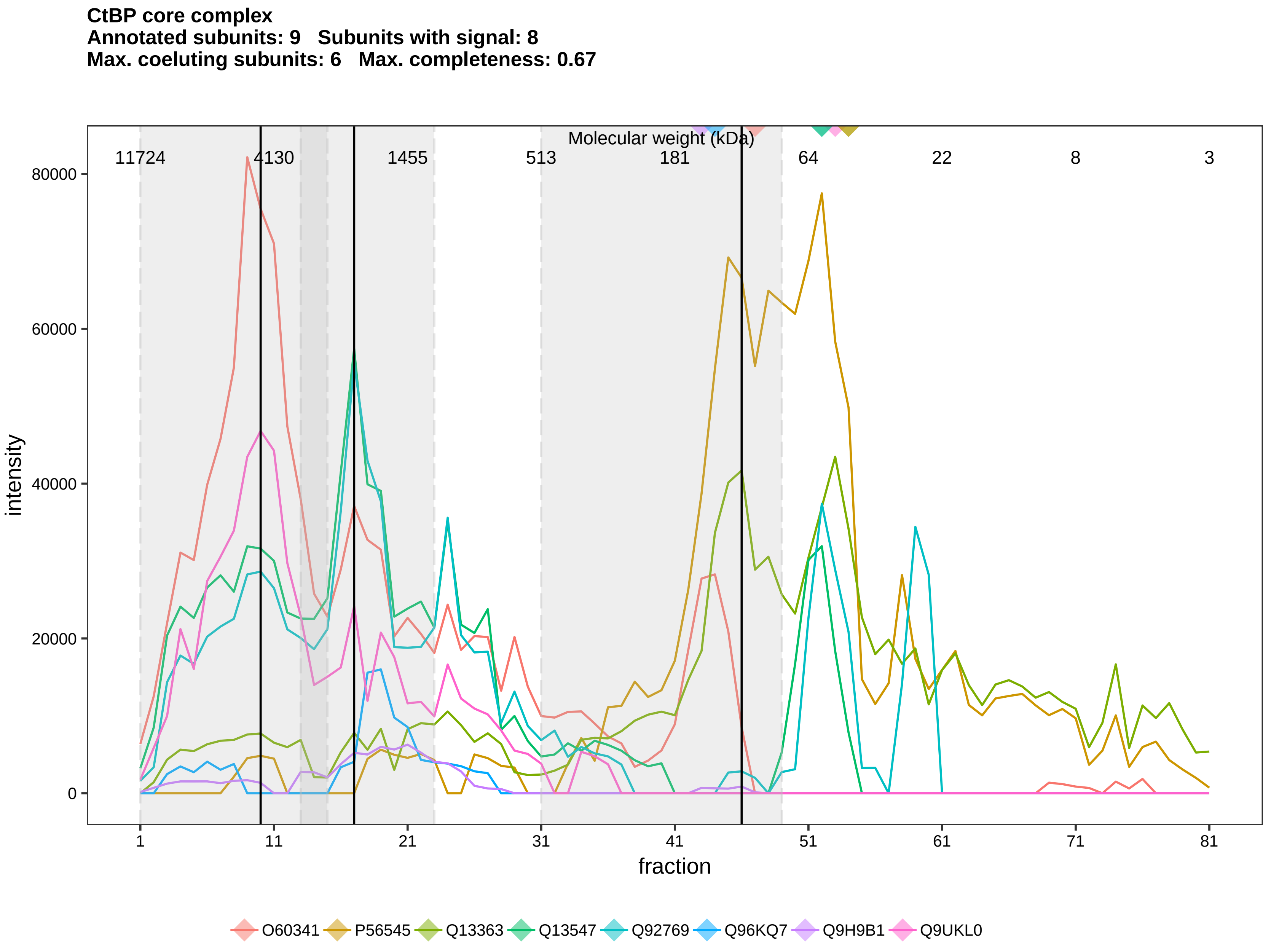

CtBP core complex
Annotated subunits: 9   Subunits with signal: 8
Max. coeluting subunits: 6   Max. completeness: 0.67
Molecular weight (kDa)
11724
4130
1455
513
181
64
22
8
3
intensity
80000
60000
40000
20000
0
1
11
21
31
41
51
61
71
81
fraction
O60341
P56545
Q13363
Q13547
Q92769
Q96KQ7
Q9H9B1
Q9UKL0
